# A possible mechanism for neurofilament slowing down in myelinated axon: Phosphorylation-induced variation of NF kinetics

**DOI:** 10.1371/journal.pone.0247656

**Published:** 2021-03-12

**Authors:** Zelin Jia, Yinyun Li

**Affiliations:** School of Systems Science, Beijing Normal University, Beijing, China; Aix Marseille University, FRANCE

## Abstract

Neurofilaments(NFs) are the most abundant intermediate filaments that make up the inner volume of axon, with possible phosphorylation on their side arms, and their slow axonal transport by molecular motors along microtubule tracks in a “stop-and-go” manner with rapid, intermittent and bidirectional motion. The kinetics of NFs and morphology of axon are dramatically different between myelinate internode and unmyelinated node of Ranvier. The NFs in the node transport as 7.6 times faster as in the internode, and the distribution of NFs population in the internode is 7.6 folds as much as in the node of Ranvier. We hypothesize that the phosphorylation of NFs could reduce the on-track rate and slow down their transport velocity in the internode. By modifying the ‘6-state’ model with (a) an extra phosphorylation kinetics to each six state and (b) construction a new ‘8-state’ model in which NFs at off-track can be phosphorylated and have smaller on-track rate, our model and simulation demonstrate that the phosphorylation-induced decrease of on-track rate could slow down the NFs average velocity and increase the axonal caliber. The degree of phosphorylation may indicate the extent of velocity reduction. The Continuity equation used in our paper predicts that the ratio of NFs population is inverse proportional to the ratios of average velocity of NFs between node of Ranvier and internode. We speculate that the myelination of axon could increase the level of phosphorylation of NF side arms, and decrease the possibility of NFs to get on-track of microtubules, therefore slow down their transport velocity. In summary, our work provides a potential mechanism for understanding the phosphorylation kinetics of NFs in regulating their transport and morphology of axon in myelinated axons, and the different kinetics of NFs between node and internode.

## 1 Introduction

Neurofilaments (NFs) are the most abundant intermediate filaments in axons of mature neurons, their population inside the axon may shape the morphology of axon [1–6]. NFs are transported by molecular motors of kinesin and dynein along microtubule tracks at slow velocities, which is named “slow axonal transport” [5, 7–12]. It is observed by fluorescence photo bleaching technique that the NFs transported through the bleached zone in a fast, intermittent and highly asynchronous manner [13–17]. Brown and Jung proposed a transport model of NF kinetic in axons: ‘6-state’ model [18]. According to ‘6-state’ model, a Gaussian wave can be generated that was highly matched with experimental data, and the transporting waves could be completely described by mean and variance [18].

It was observed that NF transport in myelinated axon decreased which induced a fatter axonal caliber in experimental observations [3, 4, 19–24]. In non-myelinated axon where the caliber is much thinner, NFs average velocity is much higher than it in the myelinated axon [19]. More recently, ex-vivo experiment were carried out and it was found that the axonal caliber in the internode can be 7.6 folds as large as it in the node of Ranvier, where the average velocity of NFs is correspondingly 7.6 folds as fast as that in the internode [19]. There are several factors that may slow NFs down in the internode, for example, myelination of axon and phosphorylation of NFs side arms. As NFs comprise light, middle and heavy chains (NF-L, NF-M and NF-H), and NF-H and NF-M have distinctively long carboxyl-terminal domains that become highly phosphorylated after newly formed NFs enter the axon [22, 24–28]. Experimental evidence showed that the NFs phosphorylation may slow down NF transport [1, 29–31]. For instance, a paper showed that C-terminal phosphorylation of NF-H gradually limits the association of NF with kinesin [22, 32]. Another paper showed that myelinating axons may selectively phosphorylate NFs and control NFs accumulation [27]. Besides, Thomas B. Shea and his colleagues demonstrated that NFs phosphorylation fosters NF-NF associations that compete with axonal transport by C-terminal phosphorylation [32, 33]. However, others showed that the average transport velocity of NFs was not changed by the NF phosphorylation [34–37]. Our work attempts to interpret the effect of phosphorylation on the kinetics of NFs specifically by comparing the NFs kinetics between the myelinated internode and unmyelinated node of Ranvier.

In this work, the kinetics of phosphorylation of NFs are added into the ‘6-state’ model of NFs transportation, and a new ‘8-state’ model is developed based on the kinetics of phosphorylation and dephosphorylating of NFs. We construct a model of single axon with both internode and node of Ranvier, and propose a possible mechanism for understanding the slowing down of NFs at myelinated internode meanwhile velocity acceleration at unmyelinated node of Ranvier, and morphological difference between node and internode. Our results demonstrate that NF kinetics of getting on-track based on the phosphorylation state regulates the average velocity of NFs and thus shape the morphology of axon.

The organization of the paper is shown in the following. In the result section 2.1, the result from modification of the ‘6-state’ by adding phosphorylation kinetics is shown; in section 2.2, the result of new ‘8-state’ model is shown to demonstrate the regulation of phosphorylation in NF kinetics and morphology of axon, and the result of NF kinetic difference and morphological difference of axon are shown by reconstruction of on-track rate in node and internodes in both PDE solution and Monte Carlo simulation. In section 3, conclusion and discussion are presented. In section 4 of model and methods, in section 4.1, the original ‘6-state’ model [18] is described and kinetics of phosphorylation and dephosphorylation has been put into each six state to explain the regulation effect of phosphorylation on NF velocity difference between node and internode, In section 4.2, a newly developed ‘8-state’ model is presented, and the expression for average velocity is derived; In addition, the strategy of modulating on-track rate is described.

## 2 Results

### 2.1 The analytical and numerical result for ‘6-state’ model with phosphorylation kinetics

#### 2.1.1 The analytical calculation for the NF velocity with phosphorylation kinetics in each six states

We use mathematical analysis to predict the velocity of NFs at node and internode. When NFs transport reach dynamically equilibrium state, and it leads to: $\left\{ \begin{array}{c} \frac{\partial P_{ph}}{\partial t}=-\gamma_{de}P_{ph}+\gamma_{ph}P_{de}=0\\ \frac{\partial P_{de}}{\partial t}=+\gamma_{de}P_{ph}-\gamma_{ph}P_{de}=0 \end{array}\right.$

Therefore, the ratio of the probability of NFs at phosphorylated and dephosphorylated states is *P*_*ph*_: *P*_*de*_ = *γ*_*de*_: *γ*_*ph*_.

The final average velocity of NFs is contributed by two parts: one is the velocity of NFs at the phosphorylated state, and the other is the velocity of NFs at the dephosphorylated state. Therefore, an equation can be written as the summation of the following two terms as in Eq (1): the first term is the product of the fraction of NFs at dephosphorylated state $f_{de}=\frac{P_{de}}{P_{ph}+P_{de}}$ with the average velocity at the dephosphorylated state ${\overset{-}{v}}_{fast}$; the second term is the product of the fraction of NFs at phosphorylated state $f_{ph}=\frac{P_{ph}}{P_{ph}+P_{de}}$, therefore the average velocity $\overset{-}{v}$ can be written as:

$$\bar{\text{V}}={\bar{v}}_{fast}\times f_{de}+\bar{v}\times f_{ph}={\bar{v}}_{fast}\times \frac{\gamma_{de}}{\gamma_{de}+\gamma_{ph}}+\bar{v}\times \frac{\gamma_{ph}}{\gamma_{ph}+\gamma_{de}}$$ (1)

In the following, we can calculate the average velocity in both node and internode sections along the axon. The probability ratio between dephosphorylation and phosphorylation is set as:

$${P_{de}:P_{ph}=\gamma }_{de}:\gamma_{ph}=\left\{ \begin{array}{c} 1:8,\;x\leq 20\mu m\;and\;x\geq 30\;\mu m,\;internode\\ 8:1,\;x\in [20,30]\mu m,\;node\;of\;Ranvier\; \end{array}\right.$$ (1a)

Then the average velocity at both node and internode can be calculated as:

$${\overset{-}{V}}_{node}={\overset{-}{v}}_{fast}\frac{\gamma_{de}(x)}{\gamma_{de}+\gamma_{ph}}+\overset{-}{v}\frac{\gamma_{ph}(x)}{\gamma_{de}+\gamma_{ph}}=(7.6*\frac{8}{9}+1*\frac{1}{9})\overset{-}{v}=\frac{61.8}{9}\overset{-}{v}$$

$${\overset{-}{V}}_{internode}={\overset{-}{v}}_{fast}\frac{\gamma_{de}(x)}{\gamma_{de}+\gamma_{ph}}+\overset{-}{v}\frac{\gamma_{ph\;}(x)}{\gamma_{de}+\gamma_{ph}}=(7.6*\frac{1}{9}+1*\frac{8}{9})\overset{-}{v}=\frac{15.6}{9}\overset{-}{v}$$

Where, ${\overset{-}{v}}_{fast}=7.6\overset{-}{v},\;\frac{v_{node}}{v_{internode}}=\frac{61.8}{15.6}=3.96$.

**Fig 1:**
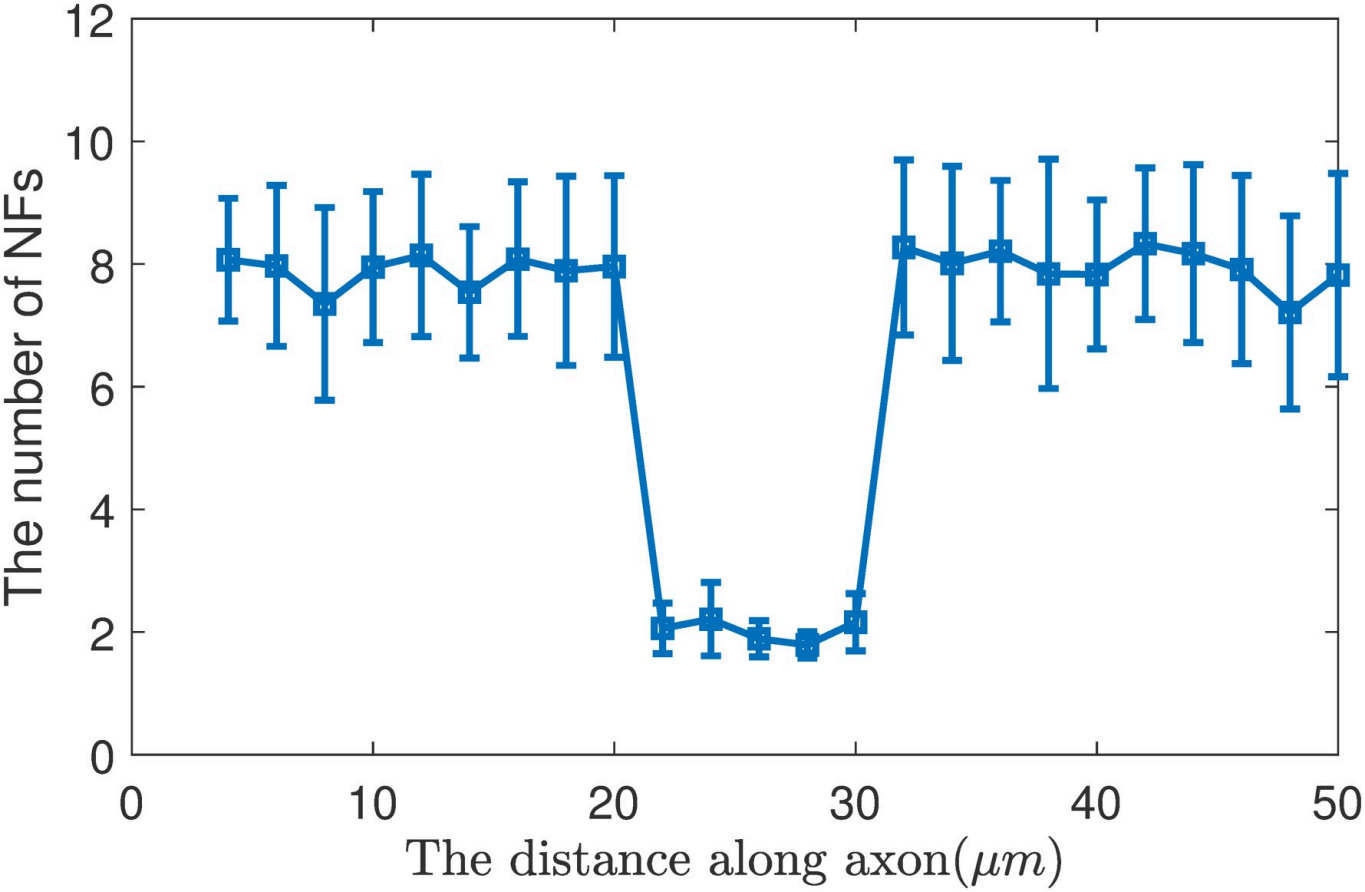
Distribution of NFs along axon in the ‘6-state’ model with kinetics of phosphorylation and dephosphorylation.

This is the theoretic prediction. Our theoretical calculation successfully predicts the simulation result as shown in Fig 1. This result is based on the experimental results of ${\overset{-}{v}}_{fast}=7.6\overset{-}{v}$ as shown in [19], it can be generalized to other type of nerves or species according to our model in Eq (1). In addition, the rate of *γ*_de_: *γ*_*ph*_ could be different for different nerves or species, therefore, the result can also be different according to Eq (1). This model is a general working frame that could be applied to different situations.

#### 2.1.2 Monte Carlo simulation with phosphorylation kinetics in each state of ‘6-state’ model

To simulate the effects of phosphorylation on the transport dynamics of NFs and axonal morphology, we construct a single axon with 50 μm long and the section of node of Ranvier is from 20 μm and 30 μm. The length of node of Ranvier ranges around 1 or 2 μm, here we assume that the node length is 10μm in order to illustrate the simulation results clearly; and our model and simulation are independent of the length of node, but depend on the kinetics of NFs phosphorylation and other parameters related with transport, and therefore can be applied to any type of axon or species.

We add the kinetics of phosphorylation and dephosphorylation into each state of ‘6-states’. Based on Eq (6), we set the corresponding value of *γ*_on_ in node and internode. At the initial section of the axon, we input NFs continuous into the axon (input one NF every 10s). We set the NFs at the initial of axon are at the state of running anterograde, if the displacement of the NFs is beyond 0–50 μm, we will remove it from our system. After reaching the steady state, the distribution of NFs population along the axon and their kinetic states are observed and recorded.

To improve the accuracy of simulation, the experiment was repeated 300 times. And we count the number of NFs in each position of the 300 axons, and calculate their mean value and the standard deviation (Fig 1).

In our simulation, we recorded each NF location and state information. Fig 1 shows the number of NFs at each position of the axon. We divided the 50 μm axon into 25 bins, where each bin is 2 μm long. We collected the number of NFs in each bin, and obtain the distribution of NFs in Fig 1. Besides, we can get the probability of NFs in each state along the axon.

According to continuity equation, $flux=N\times \overset{-}{V}$, the flux of the continuously input NFs is calculated by the product of the number of NFs (N) with the average motion velocity ($\overset{-}{V}$). Therefore, when the NF transport reaches steady state. The equation of $N_{node}\times {\overset{-}{V}}_{node}=N_{internode}\times {\overset{-}{V}}_{internode}$ will be met. Based on this, we can test our simulation results.

In order to test our simulation results, we can calculate the average velocity of NFs in the Monte Carlo simulation by the following formula:

$$\overset{-}{V}=p_{a}v_{a}+p_{r}v_{r}$$ (1b)

According to the results of Fig 1 and Eq (1b), we calculate the average velocity of NFs in the internode ${\overset{-}{V}}_{1}=2.2\;mm/day$ and in the Node of Ranvier ${\overset{-}{V}}_{2}=8.7\;mm/day$ and $\frac{{\overset{-}{V}}_{2}}{{\overset{-}{V}}_{1}}=3.95$. This is simulation result, which is close to theoretical result of 3.96.

We can obtain the average number of NFs in internode section N_1_ and in the node section N_2_ by counting the number of NFs in each position along the axon in Fig 1, and the result is $\frac{N_{1}}{N_{2}}=\frac{7.9}{2,0}=3.95$.

Remarkably, it can be seen that $\frac{{\overset{-}{V}}_{2}}{{\overset{-}{V}}_{1}}=\frac{N_{1}}{N_{2}}=3.95$. Therefore, we conclude that the continuity equation still holds true in this transport system, and we have the flux of NFs in the internode and nodes equals to each other: $N_{1}\times {\overset{-}{V}}_{1}=N_{2}\times {\overset{-}{V}}_{2}$.

#### 2.1.3 The velocity modulation depends on the rate ratio between phosphorylation and dephosphorylation r

The consistence of theoretical analysis and computational simulation has several indications.

The first indication is that, if the velocity ratio between node and internode has to be 7.6, then how much modulation of *γ*_on_ has to be made? Given the relation and distribution of phosphorylation and dephosphorylation unchanged. Based on Eq (1), the reconstruction calculation can be done in the following.

We assume ${\overset{-}{v}}_{fast}=n\overset{-}{v}$, where n is the number unknown and needs to find out, according to Eq (1):

$${\overset{-}{V}}_{internode}:{\overset{-}{V}}_{node}=\overset{-}{v}{\left(\frac{n\gamma_{de}+\gamma_{ph}}{\gamma_{de}+\gamma_{ph}}\right)}_{internode}:\;\overset{-}{v}{\left(\frac{n\gamma_{de}+\gamma_{ph}}{\gamma_{de}+\gamma_{ph}}\right)}_{node}=1:7.6$$

If Eq (1a) stands, then it will lead to ${\overset{-}{V}}_{node}:{\overset{-}{V}}_{internode}=\frac{8n+1}{n+8}=7.6$, subsequently this results in *n* = 149.5. This calculation indicates that if a velocity ratio between node and internode needs to be 7.6, the velocity of fast transport has to be boosted by 149.5 folds, i.e. ${\overset{-}{v}}_{fast}=149.5\;\overset{-}{v}$. According to construction of the on-track rates based on Eq (6) in the model and method section, the on-track rate can be calculated out.

In addition, the ratios of average velocity between internode and node can be written as functions of *n*, q_4_, where $q_{4}=\frac{\gamma_{de}}{\gamma_{ph}}$,

$${\overset{-}{V}}_{internode}:{\overset{-}{V}}_{node}={\frac{nq_{4}^{inter}+1}{1+q_{4}^{inter}}}_{\;}:\;\frac{nq_{4}^{node}+1}{1+q_{4}^{node}}$$ (1c)

If it is set $r=\frac{\gamma_{ph}}{\gamma_{de}}(internode)$, and assume in the node: $\frac{\gamma_{ph}}{\gamma_{de}}=\frac{1}{r}$, then we have:

$${\overset{-}{V}}_{node}:{\overset{-}{V}}_{internode}={\frac{nr+1}{r+1}}_{\;}:\frac{r+n}{r+1}=\frac{nr+1}{r+n}$$

**Fig 2:**
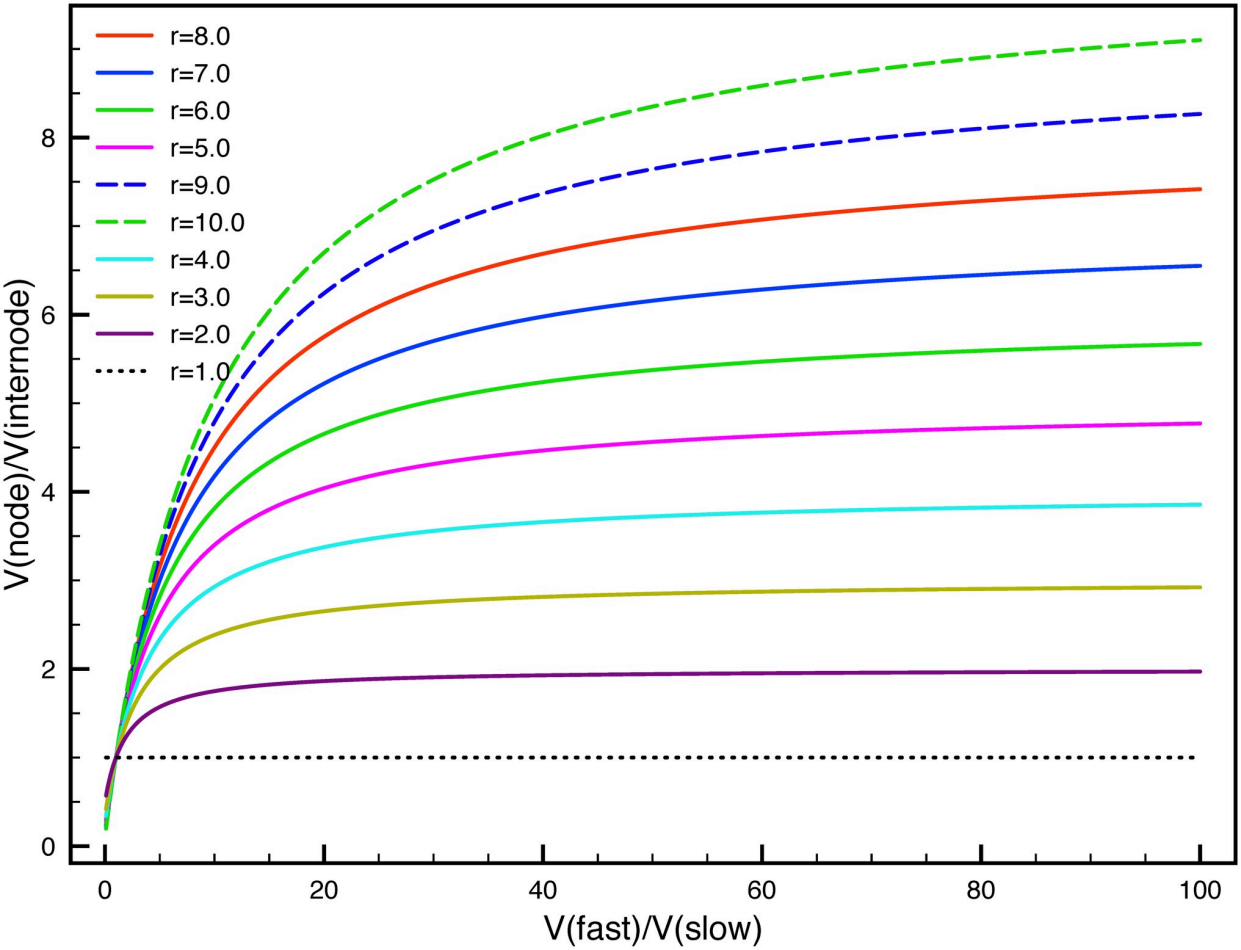
The dependence of velocity boost on the rate ratio between phosphorylation and dephosphorylation r = *γ*_ph_: *γ*_*de*_. The x-axis is the velocity ratio n between the node V(fast) and internode V(slow) without the phosphorylation kinetics. The y-axis is the velocity ratio between node V(node) and internode V(internode) with the phosphorylation kinetics.

According to Eq (1c), the ratio of velocity depends on n and *r* values in the node and internode. Therefore, if the two factors can be measured in experiments, then the average velocity between internode and node can also be predicted. We plot the velocity ratio between node and internode as function of n in Fig 2, where x axis is the velocity ratio n without the influence of phosphorylation kinetics, r is the ratio between phosphorylation rate and the dephosphorylation in the internode section. We assume in the node the ratio is inverse of it in the internode section, which is 1/r.

For different r values, the colored curve show different dependence of y on the n and r, the velocity ratio between node and internode is increasing with r = *γ*_ph_: *γ*_*de*_ for a fixed value of x. As is shown in the red curve (r = 8.0) in Fig 2, the x axis is only for 1 to100 but not reaches 149.5, but trend can be predicted there that for x = 149.5, the y axis can only reaches 7.6. In another word, if r = 9.0 in the dashed blue curve, the x axis would be x = 48.2 for the value of y = 7.6; if r = 10.0 in the dashed green curve, then x = 31.3 for the y = 7.6. Therefore, the ratio r needs to increase to reduce the effort of boosting velocity without phosphorylation kinetics.

The second indication is, we chose to increase *γ*_on_ in the node to make the velocity faster than the internode, and the supposed boost of velocity is 7.6 fold; while the phosphorylation kinetics interfere the ‘6-state’ model of NFs, by dividing each state into phosphorylated and dephosphorylated states, with different transition rates in the node and internode, the originally constructed velocity ratio between node and internode of 7.6 drops to be only 3.96. In another word, it demonstrates that the phosphorylation kinetics has decreased the influence of on-track rate *γ*_*on*_ in determining the average velocity ratio of NFs between node and internode.

As can be seen in Fig 2, for all values of r, the curves are all going to be saturated at different values for extremely large x. It indicates that no matter how fast the velocity in the node is boosted compared to the internode, with a certain phosphorylation kinetics added into the system, the velocity ratios between node and internode will eventually approaches a saturated value, which is much smaller than the value n without phosphorylation kinetics. The higher values of r, the higher the saturated ratio is going to be. In general, for all values of r, the y axis is still below the y = x curve;, which indicates that the phosphorylation kinetics has dramatically decreased the velocity ratios between node and internode with the phosphorylation kinetics added into the system.

The last point is that, as the ratio r changes, the velocity ratio between the node and internode will be regulated. We take an extreme example to illustrate the effect. If r = *γ*_*ph*_: *γ*_de_ = ∞ in the internode, then the average velocity will become ${\overset{-}{V}}_{internode}\;\approx {\overset{-}{v}}_{fast}\times 0+\overset{-}{v}\times 1=\overset{-}{v}$, according to Eq (1). On the other hand, if *γ*_ph_ ≪ *γ*_de_ in the node, then the average velocity will become ${\overset{-}{V}}_{node}\;\approx {\overset{-}{v}}_{fast}\times 1+\overset{-}{v}\times 0={\overset{-}{v}}_{fast}$. In summary, we can see that the at the limit of extremely phosphorylation in the internode and no phosphorylation in the node, the velocity ratio will become to be ${\overset{-}{V}}_{internode}:{\overset{-}{V}}_{node}\approx \overset{-}{v}:\;{\overset{-}{v}}_{fast}=1:n$. In the other extreme, if the phosphorylation ratios r = *γ*_*ph*_: *γ*_de_ = 1 in the internode, then the average velocity will also be the same between node and internode ${\overset{-}{V}}_{internode}:{\overset{-}{V}}_{node}=1:1$.

### 2.2 Simulation result of ‘8-state’ model

#### 2.2.1 Simulation result of reconstruction of velocity in ‘8-state’ model by numerical simulation of PDE

The partial differential equations in Eq (8) are discretized into ordinary differential equation by First Order Upwind to obtain numerical solution. The on-track rates reconstructed in Fig 8 are put into the simulation. In the simulation, NFs are injected into the proximal section of axon with total 50 μm long. The boundary conditions are set as: P_*_ (*x*_0_, *t*) = 0; P_*_ (*x*_*l*_, *t*) = P_*_ (*x*_*l*_ − 1, *t*); *x*_0_ and *x*_*l*_ denotes the proximal boundary axon and the distal boundary of axon, P_*_ denotes each probability of eight states. Initial condition of the PDE of NFs distribution is set by the equilibrium distribution according to Eq (9).

**Fig 3:**
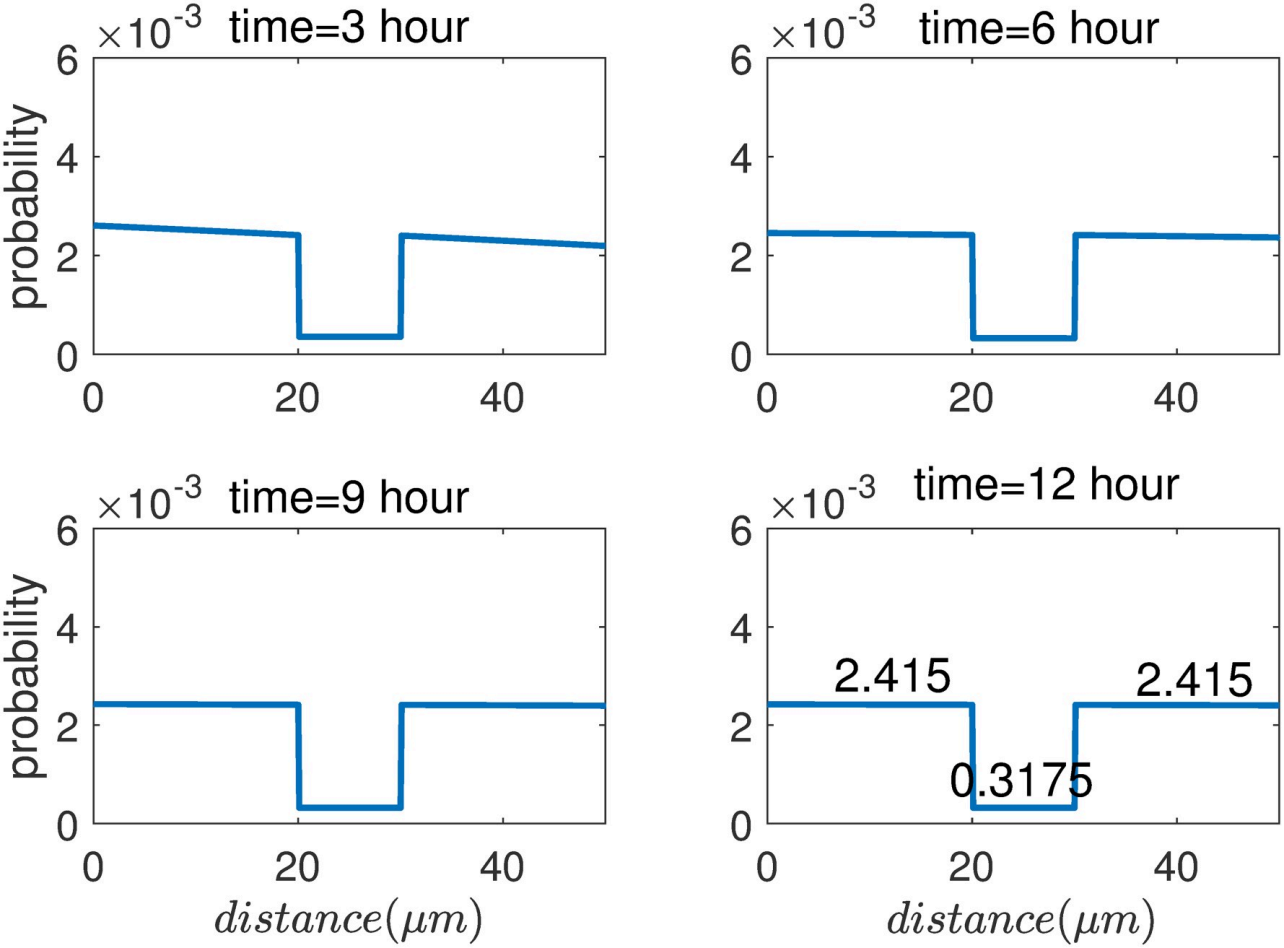
The probability of NFs at each state by reconstruction of velocity along node and internode by numerical solution of PDE.

In order to observe the distribution of NFs along the axon in the equilibrium state, NFs are continuously input into the axons from the proximal section (input one unit of NF population every 1s). The results of the numerical simulation are shown in the Fig 3. As can be seen, the NFs distribution along axon does not change after 9 hours, reaching equilibrium state. Note that it is a dynamical equilibrium, with constant flux of NFs entering into the proximal axon and the same constant flux exiting axon at the distal section.

As can be seen in Fig 3, the ratio of population of NFs between internode and node of Ranvier is 2.415:0.3175 = 7.6. Note that the section form 20 μm to 30 μm is the node of Ranvier. It not only reproduces experimental observation results [6], but also consists with the theoretical prediction in Sec.4.2.1.

**Fig 4:**
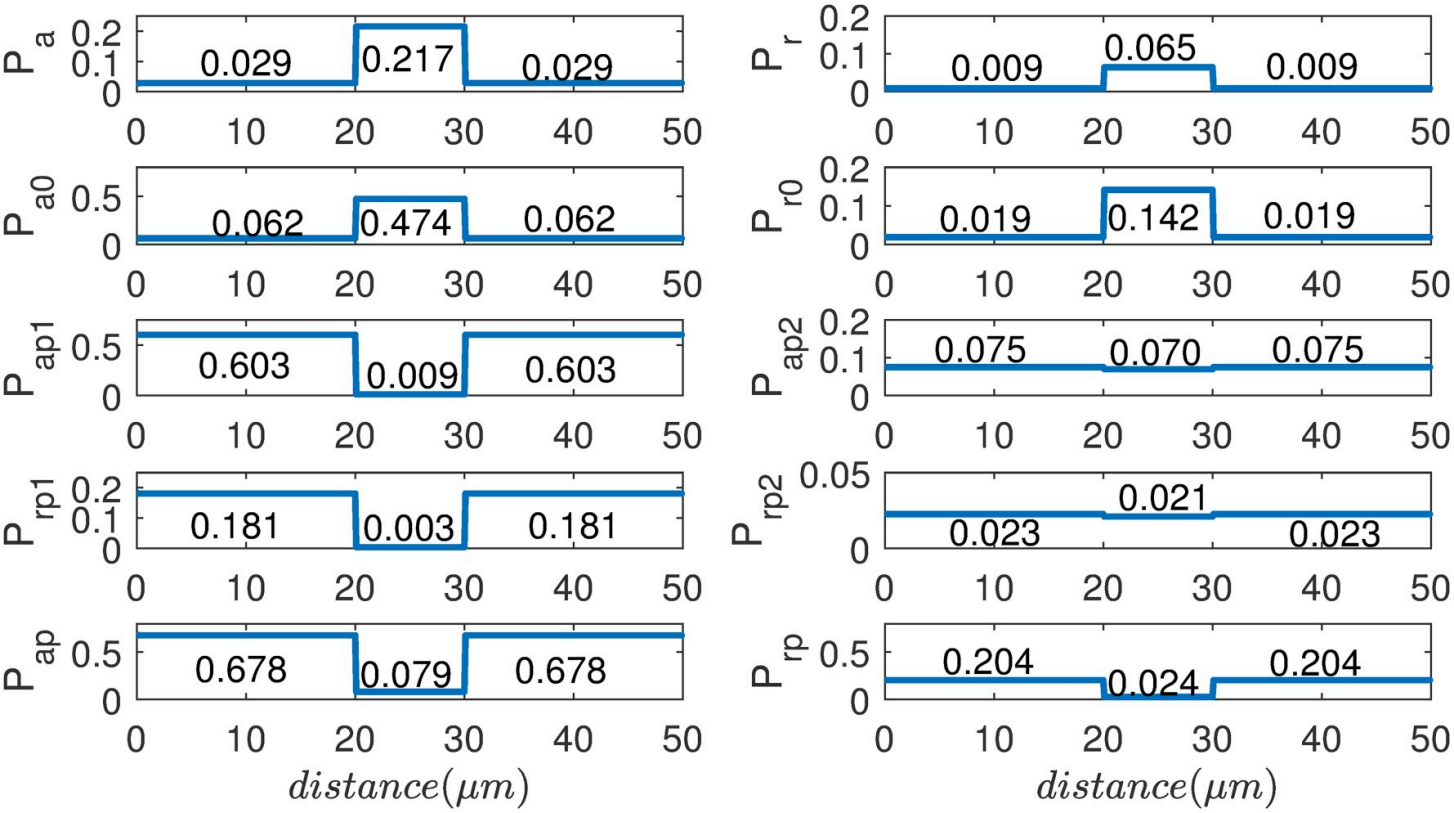
The distribution of NFs in each state at dynamical equilibrium by velocity reconstruction in the ‘8-state’ model.

As can be seen from Fig 4, the probability of NFs at each eight state is differentially distributed along the axon. When the NFs enter the node of Ranvier, due to the higher dephosphorylation rate and thus higher on-track rate, probabilities of NFs at on-track states *P*_a_, *P*_*a*0_, *P*_*r*_, *P*_*r*0_ increase, while the probability of *P*_*ap*_, P_rp_ decrease. At the node of Ranvier, the NFs at off-track states are dephosphorylated, thus NFs at P_ap1_, *P*_*rp*1_ are much lower; while NFs in the internode are highly phosphorylated in the axon, thus the NFs at states of P_ap1_, *P*_*rp*1_ are much higher in the internode. Therefore, the introduction of transition kinetics with different rates of phosphorylation and dephosphorylation in node and internode, and its modulation on the “on-track” rate $\gamma_{on}^{2}$ can eventually demonstrate the influence of phosphorylation on NF transport.

#### 2.2.2 The Monte Carlo simulation result of reproduction of velocity in ‘8-state’ model

**Fig 5:**
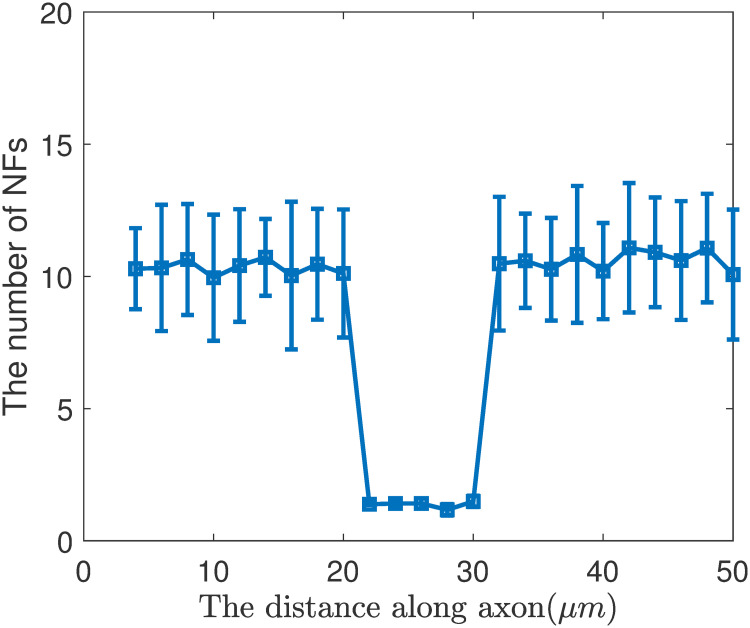
Distribution of NFs along axon by reconstruction of on-track rate in the ‘8-state’ model.

We use Monte Carlo method to simulate the ‘8-state’ model, the value of *γ*_de_ and *γ*_ph_ are shown in Fig 9 and *$\gamma_{on}^{2}$* are shown in Fig 11 in the section of model and methods. At the initial section, NFs are continuously injected into the axon (input one NF every 10 seconds). To improve the accuracy of simulation, the experiment was repeated 300 times. The number of NFs in each bin of the axon (2μm) is counted for all the 300 axons, each NF’s state are recorded and their mean values and the standard deviation are calculated. The simulation result is shown in Fig 5.

According to the results of Fig 5 and Eq (1b), we calculate the average velocity of NFs in the internode, ${\overset{-}{V}}_{internode}=p_{a2}\times v_{a}+p_{r2}\times v_{r}=0.018\frac{\mu m}{s}$ and in the node of Ranvier, ${\overset{-}{V}}_{node}=p_{a1}\times v_{a}+p_{r1}\times v_{r}=0.132\frac{\mu m}{s}$, and we have $\frac{{\overset{-}{V}}_{node}}{{\overset{-}{V}}_{internode}}=7.33$ which is close to 7.6.

From the simulation result of Fig 5, we can count the average number of NFs in the internode *N*_1_ = 10.82 and node of Ranvier N_2_ = 1.44 and we have $\frac{N_{1}}{N_{2}}=\frac{10.48}{1.37}=7.51$, which is close to the inverse ratio of average velocity: $\frac{N_{1}}{N_{2}}=\frac{{\overset{-}{V}}_{2}}{{\overset{-}{V}}_{1}}=7.6$. Therefore, the reconstruction is valid by holding the continuity equation true along the same axon of node and internode sections. $N_{1}{\times \overset{-}{V}}_{1}=N_{2}{\times \overset{-}{V}}_{2}$. In addition, this reconstruction fully considered the phosphorylation induced on-track rate change, and the simulation results from PDE and Monte Carlo method are the same. Our method successfully reproduces the experimentally observed NFs population distribution along axon. Our model provides a possible mechanism for understanding the NFs slowing down in the myelinated internode.

#### 2.2.3 Analysis of the on-track rate $\gamma_{on}^{2}$ and phosphorylation rate ratio r

**Fig 6:**
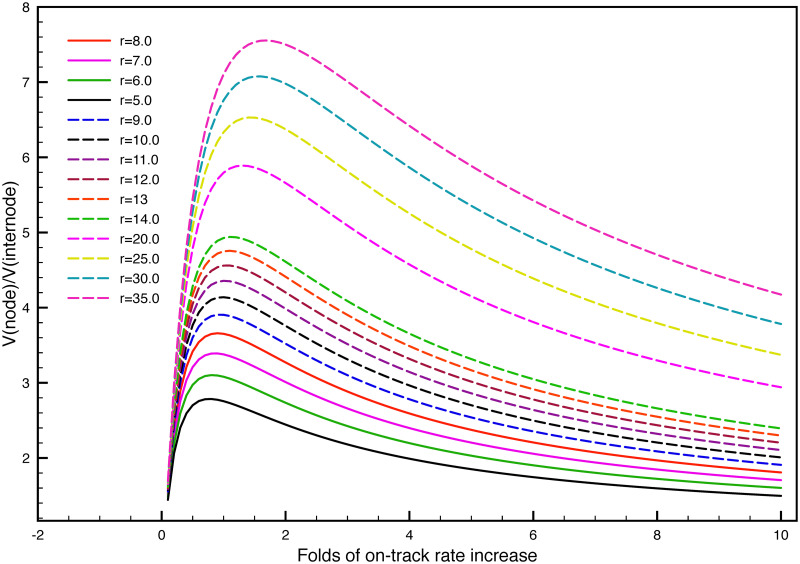
The velocity acceleration in the node depends on the parameter of r and increase folds of $\gamma_{on}^{2}$. The x axis are the increase folds of the on-track rate $\gamma_{on}^{2}$ for both the node and internode, increasing from $\gamma_{on}^{2}=0.00498\;s^{-1}$ to $\gamma_{on}^{2}=0.0498\;s^{-1}$ by ten folds.

**Fig 7:**
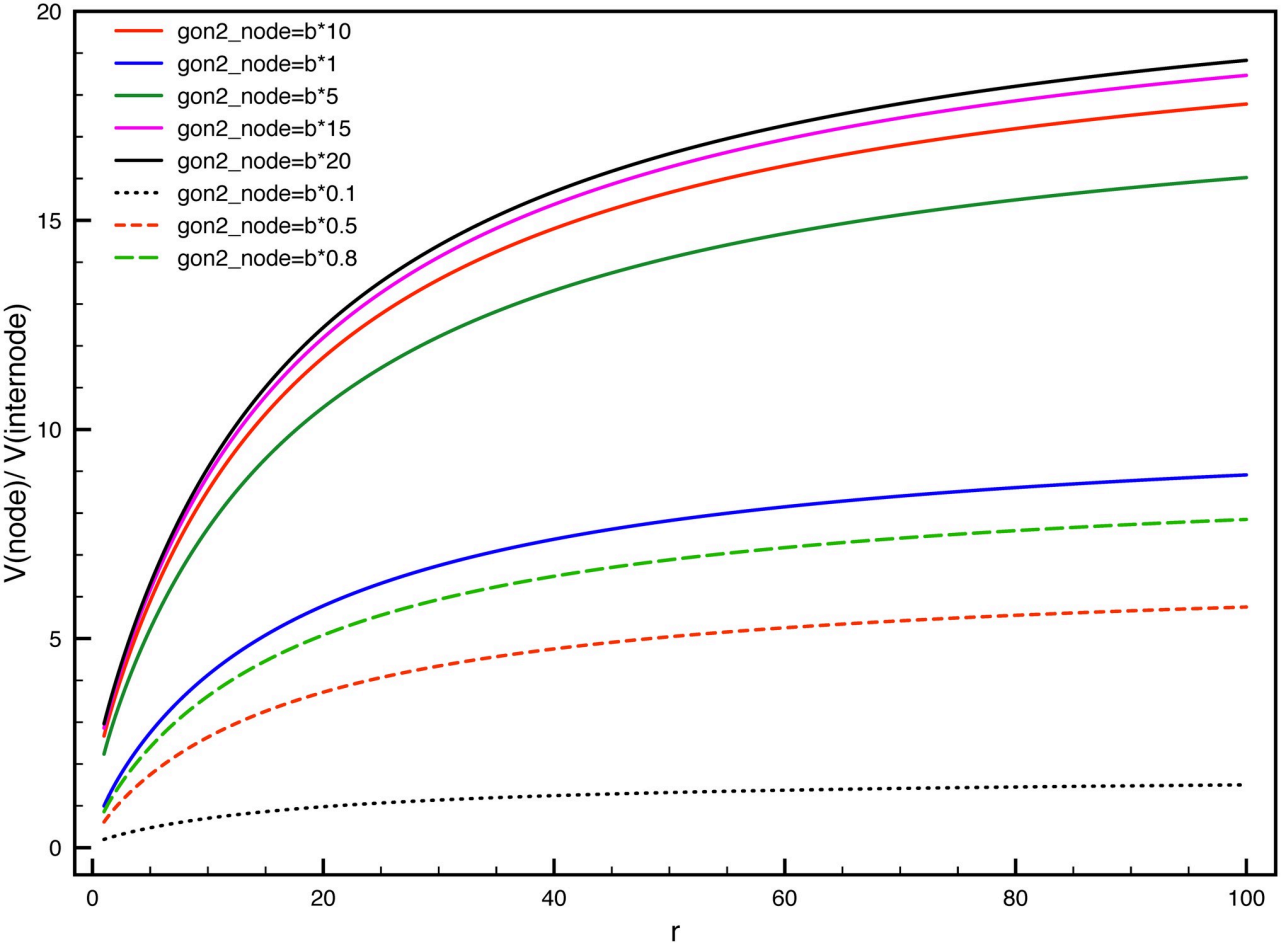
The ratio of velocity between node and internode changes with the rate ratio of phospohorylation and dephosphorylatoin r = γ_ph_: γ_*de*_ in the internode. The $\gamma_{on}^{2}$ in the internode is fixe as b = 0.00498 s^−1^.

If the rate ratio between phosphorylation and dephosphorylation in the internode r = γ_ph_: *γ*_*de*_ (and the ration will be 1/r at node of Ranvier) varies, and the on-track rate $\gamma_{on}^{2}$ changes simultaneously for both internode and node, while the rate $\gamma_{on}^{1}=0.000275s^{-1}$ is unchanged, then it can be seen how the velocity ratio between node and internode changes with the parameter r in Fig 6. The larger r value is, the higher velocity boost in the node will be for a certain on-track rate $\gamma_{on}^{2}$. For one certain value of r, there is always an optimized value of $\gamma_{on}^{2}$ to maximize the velocity ratio in the y axis. In order to reach a desired value in y axis (boost the velocity in the node), it may not work by simply increasing $\gamma_{on}^{2}$, the value of $\gamma_{on}^{2}$ has to cooperate together with parameter r to reach the desired velocity acceleration in the node. Another point from Fig 6 is that, if $\gamma_{on}^{2}$ is the same for node and internode, then it is not possible to reach the desired velocity ratio in the y axis for a chosen r. For example, if r = 8 in the red solid curve, and no matter how much variation of $\gamma_{on}^{2}$ has, it is not possible to reach a velocity ratio 7.6. In Fig 7, for the case of same on-track values $\gamma_{on}^{2}$ for both node and internode, as is shown in the blue curve(gon2_node = b*1), the value of r has to be more than 30.0. to reach the velocity ratio of 7.6 in y axis.

On the other hand, as the ratio of on-track rate $\gamma_{on}^{2}$ between node and internode increases, the velocity ratio between node and internode can be boosted for different values of r, as can be seen in Fig 7. Another point can be seen in Fig 7 is that, when the $\gamma_{on}^{2}$ in the node is smaller than the internode, the velocity ratio can also be larger than one if the r value is selected poroperly. For example, for the dashed red curve, the $\gamma_{on}^{2}$ value in the node is half of it in the internode, the y axis can still be larger than 1 and can reach 5 for larger r values. This indicates that the phosphorylation kinetics itself can balance the lower on-track rate in the node and still result in a faster velocity in the node of Ranvier.

## 3 Conclusion and discussion

### 3.1 Conclusion

Based on the “stop-and-go” hypothesis, firstly we tried to add phosphorylation kinetics in each state, and analyzed the effect of phosphorylation on the average velocity of NFs transport in the node and internode. The basic assumption is the on-track rate is modulated by the phosphorylation kinetics, and NF transport velocity and the distribution of NFs population are thus changed by the on-track rate, according to the continuity equation. Secondly, by assuming that only at off-track states, NFs have phosphorylation kinetics and the phosphorylation status will eventually regulate the on-track rate, in which we introduce the kinetic transition between phosphorylation and dephosphorylation of NFs and build the ‘8-state’ model. Our model demonstrated that the regulation of phosphorylation on NF transport and axon morphology, and provided a potential mechanism for NFs slowing down during myelination.

The modulation of the on-track rates of NFs is based on the assumption that NFs in node of Ranvier are less phosphorylated thus the on-track rate is higher, while in the internode, due to highly phosphorylation, NFs on-track rate is lower. Another basis of our modulation is that the NFs flux is a constant at equilibrium state, which results in the inverse proportional relationship between population of NFs and the average velocity distribution along node and internode. Through the Monte Carlo simulation and numerical solution of PDE of ‘8-state’ model, our results demonstrate the dephosphorylation of NFs can accelerate NFs kinetics in the node of Ranvier and phosphorylation of NFs can decrease NFs kinetics in the internode. In the end, our simulation results show that ratio of the number of NFs is inverse proportional to the average of velocity of NFs at both node and internode. Our model provides a potential mechanism to understand the regulation of NFs kinetics at myelinated axon by NFs phosphorylation kinetics; besides, the dynamic equilibrium for the kinetics between phosphorylation and dephosphorylation determines the fraction of NFs at each state, which regulates the on-track rate of NF transport, and this mechanism can be applied to any axon for any species.

### 3.2 Discussion

However, our model still needs to be improved in the following aspects. First, we assume that the NFs are independent of each other, and do not consider the interaction between them, which is certainly unrealistic in the real axonal transport. Besides, NF-NF association results in the formation of NF "bundles", the “bundles” may have important effect on the axonal transport. Second, the transition rate constants γ_10_, *γ*_01_, *γ*_*ra*_, *γ*_*ar*_ are put into our model according to the “stop-and-go” model [18] and by the observation data of rat superior cervical ganglion neurons published by the research [19]. Noting that the node of Ranvier is relatively short compared with the internode section of axon, it must be difficult to measure the average velocity of NFs passing through the node in the experiments. When the experiment has new progress, our model can modify these parameters in according to the new experimental data.

Our model assumed different rates of phosphorylation and dephosphorylation between nodes and internodes, which have not been experimentally observed, and may have different values in different species or types of nerves. However, Data presented in [38] clearly distinguish between the densities of NF phosphorylated epitopes in internodes versus nodes of Ranvier. NF-H and NF-M phosphorylated epitopes are reduced by 60 and 40% respectively in the nodes relative to internodes. The basic principle of our model can predict transport velocities of NFs based on different values of phosphorylation rate constants. In addition, according to the literature, the phosphorylation rates of NFs may change under differential myelination level [24, 27, 39]. In this sense our model provides a possible mechanism for understanding myelination regulation of NFs transport inside the axon.

There are different results, regarding on the experimental observation of NFs transport due to the NFs phosphorylation. Some experiments show that the phosphorylation slow down the NFs transport [1, 40, 41], while others show that phosphorylation did not slow down NFs transport [34–37]. We can interpret that the level of phosphorylation and dephosphorylation in regulating the NFs on-track rate might be the key. For example, if the NFs in the internode are heavily phosphorylated, it might decrease the on-track rate severely, then the average velocity will be slowed down. On the other hand, if NFs are less phosphorylated in the internode, the on-track rate will be less affected, and the average velocity will not be slowed down. During the myelination process occurring in the developmental period, the caliber of axon gradually increases and it might be interpreted as due to the gradual phosphorylation of the NFs which decreases the on-track rates gradually, and results in the slowing down of NF transport. Therefore the phosphorylation effect can be both spatial and temporal, and the effect on the rate of on-track and NFs average transport velocity will follow, and that needs to be investigated in the future work.

## 4 Model and methods

### 4.1 Phosphorylation kinetics added to each state of ‘6-state’ model

In this section, we add NF transition kinetics of phosphorylation and dephosphorylation into each state in the ‘6-state’ model. By assuming that phosphorylated NFs have lower on-track rate and dephosphorylated NFs have higher on-track rate, we show that NFs phosphorylation regulates the average velocity of the NFs and their final distribution along axon by both analytical solution and simulation results.

#### 4.1.1 Model description of the phosphorylation kinetics added into the ‘6-state’ model

We model NFs transport based on a previous model: ‘6-state’ model [18], where each NF moves bi-directionally along the axon switching between six kinetic states (*P*_a_, *P*_*r*_, *P*_*a*0_*P*_*r*0_, *P*_*ap*_, *P*_*rp*_). The model gave an analytical expression for average velocity expression; and the model can produce simulation results in a good approximation of experimental data for the group transport wave of Gaussian function. The model mathematical expression can be written by coupled Partial Differential Equations (PDE) in Eq (2).

$$\begin{array}{ll} \frac{\partial {\text{P}}_{\text{a}}}{\partial t} & =-v_{a}\frac{\partial P_{a}}{\partial x}-\gamma_{10}P_{a}+\gamma_{01}P_{a0}\\ \frac{\partial {\text{P}}_{r}}{\partial t} & =-\frac{v_{r}\partial P_{r}}{\partial x}-\gamma_{10}P_{r}+\gamma_{01}P_{r0}\\ \frac{\partial {\text{P}}_{\text{a}0}}{\partial t} & =-(\gamma_{01}+\gamma_{ar})P_{a0}+\gamma_{10}P_{a}+\gamma_{ra}P_{r0}+\gamma_{on}P_{ap}-\gamma_{off}P_{a0}\\ \frac{\partial {\text{P}}_{\text{r}0}}{\partial t} & =-(\gamma_{01}+\gamma_{ra})P_{r0}+\gamma_{10}P_{r}+\gamma_{ar}P_{a0}+\gamma_{on}P_{rp}-\gamma_{off}P_{r0}\\ \frac{\partial {\text{P}}_{\text{ap}}}{\partial t} & =\gamma_{off}P_{a0}-\gamma_{on}P_{ap}-\gamma_{ar}P_{ap}+\gamma_{ra}P_{rp}\\ \frac{\partial {\text{P}}_{\text{rp}}}{\partial t} & =\gamma_{off}P_{r0}-\gamma_{on}P_{rp}-\gamma_{ra}P_{rp}+\gamma_{ar}P_{ap} \end{array}$$ (2)

Where *P*_a_(*x*, *t*), *P*_*r*_(*x*, *t*), *P*_*a*0_(*x*, *t*), *P*_*r*0_(*x*, *t*), *P*_*ap*_(*x*, *t*), *P*_*rp*_(*x*, *t*) represent the distribution of running-anterograde, running-retrograde, pausing-anterograde, pausing-retrograde, off-track-anterograde, and off-track-retrograde, respectively. In our simulation, the anterograde and retrograde velocities *v*_*a*_ = 0.52 *μm*/*s*, *v*_*r*_ = −0.36*μm*/*s* are adopted from research [2, 7, 8, 18, 40, 41].

The spatial distribution of NFs population is: *P*(x, t) = *P*_a_(*x*, *t*) + *P*_*r*_(*x*, *t*), *P*_*a*0_(*x*, *t*) + *P*_*r*0_(*x*, *t*) + *P*_*ap*_(*x*, *t*) + *P*_*rp*_(*x*, *t*).

If NFs enter the axon at the proximal section and leave the axon at distal section at a constant rate, after long time, the steady distribution of NFs in the kinetic states can be obtained readily by solving the above equations. The theoretical solution of probability at each of the six states are [18]:

$$p_{a}=\rho ,\;p_{r}=q_{3}\rho ,\;p_{a0}=q_{1}\rho ,\;p_{r0}=q_{1}q_{3}\rho ,\;p_{ap}=q_{1}q_{2}\rho ,\;p_{rp}=q_{1}q_{2}q_{3}\rho .$$ (3)

Where, $\rho =\frac{1}{\left(1+q_{1}(1+q_{2}))(1+q_{3}\right)};q_{1}=\frac{\gamma_{10}}{\gamma_{01}},\;q_{2}=\frac{\gamma_{off}}{\gamma_{on}},\;q_{3}=\frac{\gamma_{ar}}{\gamma_{ra}}$.

For the paper, the rate parameters are set as [18]: γ_10_ = 0.14 *s*^−1^, *γ*_01_ = 0.064 *s*^−1^, *γ*_*off*_ = 0.00445 *s*^−1^, *γ*_*ra*_ = 1.4 × 10^−5^ *s*^−1^, *γ*_*ar*_ = 4.2 × 10^−6^ *s*^−1^. The rate of on-track will be modified in the latter section.

**Fig 8:**
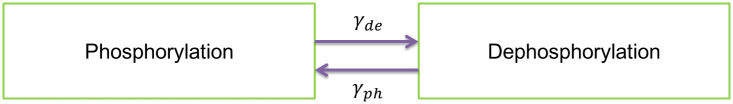
NFs kinetics between states of phosphorylation and dephosphorylation.

According to the experimental result, the average movement velocity of NFs in the node of Ranvier is 7.6 times as that in the internode sections [19]. Why the NFs can accelerate so rapidly at node of Ranvier? Here, we introduce the transition kinetics between phosphorylation and dephosphorylation into the ‘6-state’ model, and investigate how phosphorylation of NFs could regulate the kinetics of NFs. At first, we assume for each six state, NFs can have the kinetics of phosphorylation and dephosphorylation as seen in Fig 8. As NFs can have different episodes for phosphorylation either in NFH or in NFM or both [42, 43], and the NFs phosphorylation at different episodes and how many episodes are phosphorylated involves complicated chemical reactions of kinase or other proteins [44], in order to make our model concise and can be generalized to various conditions, we from biophysical perspective assume that the NFs as a one unit that can have two state of phosphorylation, and the percentage of phosphorylated or level of phosphorylation can be recognized as the fraction of NFs at phosphorylated state compared to the dephosphorylated state.

The kinetics of phosphorylation and dephosphorylation can be written in the following equation:

$$\{\begin{array}{c} \frac{\partial P_{ph}}{\partial t}=-\gamma_{de}P_{ph}+\gamma_{ph}\;P_{de}\\ \frac{\partial P_{de}}{\partial t}=-\gamma_{ph}P_{de}+\gamma_{de}P_{ph} \end{array}$$ (4)

**Fig 9:**
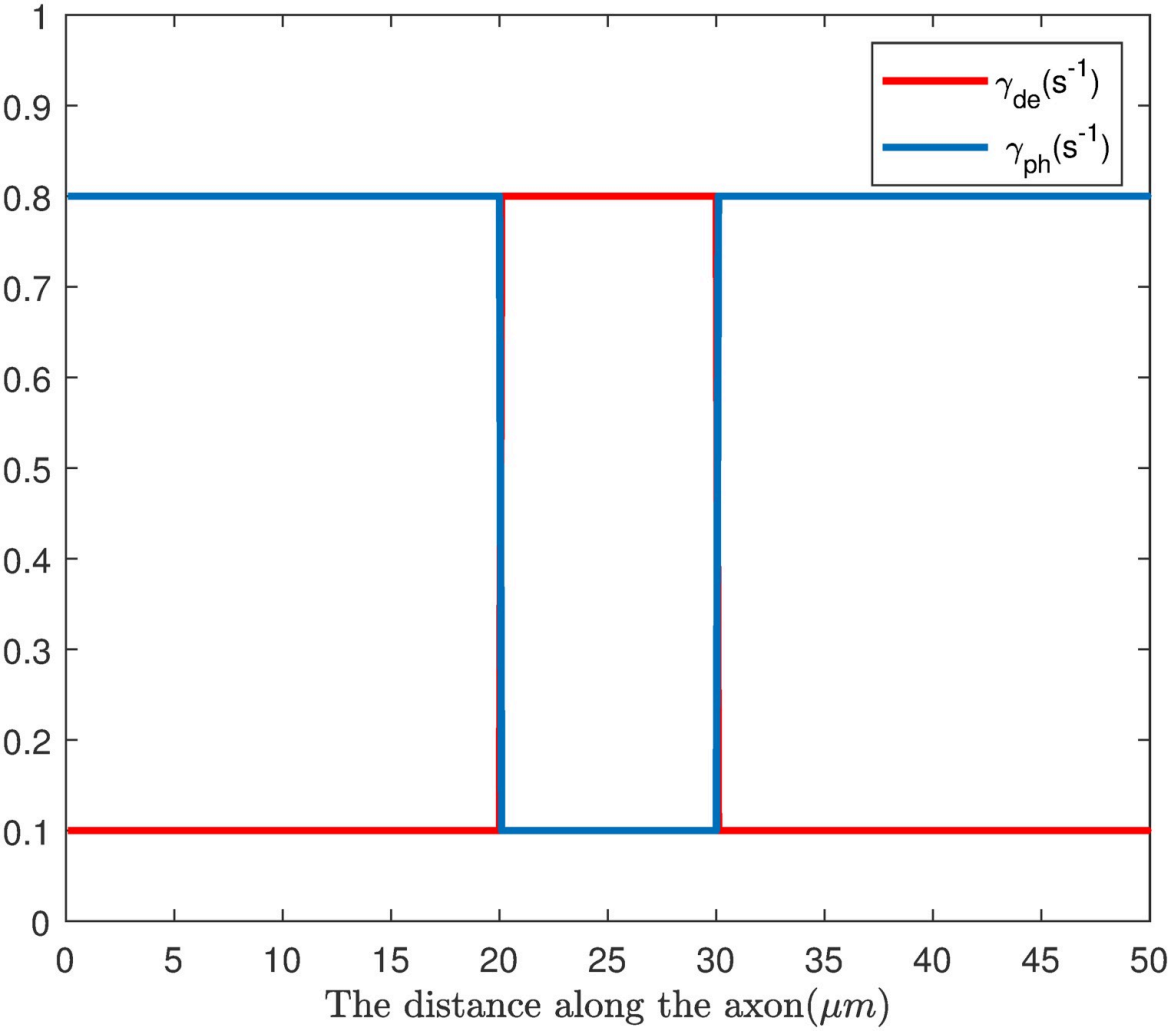
The distribution of γ_de_, *γ*_*ph*_ along the axon.

Where *P*_*ph*_(*x*, *t*) and *P*_*de*_(*x*, *t*) represent the distribution of phosphorylated and dephosphorylated NFs respectively; the rate of γ_de_ and γ_ph_ are assumed to be dependent on the location where NFs transport through, which is related to myelination function along the axon. According to the experimental observations [18, 25, 30, 32], we assume that the NFs has a higher rate of phosphorylation at internode due to the myelination process around axon, while NFs has a higher rate of dephosphorylation at node of Ranvier, where there is no myelination wrapped, as can be seen in Fig 9. The rate of phosphorylation and dephosphorylation in the internode is γ_ph_ = 0.8, *γ*_*de*_ = 0.1. while the node, the rates are set opposite: γ_ph_ = 0.1, *γ*_*de*_ = 0.8.

#### 4.1.2 Reconstruction of the on-track rate

We assume that phosphorylation of NFs makes NFs difficult to get on-track of microtubule by either tangling with neighboring NFs or affecting the interaction between the NFs and molecular motor kinesin or dynein, therefore, therefore, it decrease the rate of on-track γ_on_. On the contrary, NFs dephosphorylation can reduce side arm length, and facilitate NFs to jump on-track for running, which results in higher on-track rate. In the following part, the on-track rate reconstruction is illustrated based on the continuity equation [14].

From paper [18], the average movement velocity $\overset{-}{v}$ is:

$$\bar{v}=\frac{d<x(x,t)>}{\text{dt}}=\frac{1}{\left(1+{\text{q}}_{1}(1+{\text{q}}_{2}))(1+{\text{q}}_{3}\right)}(v_{a}+q_{3}v_{r})$$ (5)

The magnitude of $\overset{-}{v}$ depends on the coefficients q_1_, *q*_2_ and *q*_3_. The on-track rate of γ_on_ can be re-written as a function of average velocity as follows:

$$\gamma_{\text{on}}(x)=\frac{\gamma_{off}\bar{v}(x)(1+q_{3})q_{1}}{v_{a}+q_{3}v_{r}-\bar{v}(x)(1+q_{3})(1+q_{1})}$$ (6)

Based on Eq (5), with other transition parameters unchanged, if the NF average velocity in the node of Ranvier is 7.6 times as that in the internode section, then the on-track rate will become $\gamma_{on}^{'}=5.16\times 10^{-3}\;s^{-1}$ (corresponding to average velocity of ${\overset{-}{v}}_{fast}=7.6\overset{-}{v}=7.6\times \;0.71mm/day=5.31mm/day$), while average velocity in the internode, the on-track rate is only $\gamma_{on}=2.75\times 10^{-4}\;s^{-1}(\overset{-}{v}=0.71mm/day)$.

Therefore, for each six states, when NFs are at the state of phosphorylation, the on-track rate will be lower (γ_on_ = 2.75 × 10^−4^ *s*^−1^, $\overset{-}{v}=0.71\;mm/day);$ while NFs are at dephosphorylation state, their on-track rate will be higher ($\gamma_{on}^{'}=5.16\times 10^{-3}\;s^{-1}$.$\;{\overset{-}{v}}_{fast}=5.31\;mm/day$).

Thus, we set the value of γ_on_ to be location dependent as follows:

$$\gamma_{\text{on}}=\{\begin{array}{ll} \gamma_{on}=2.75\times 10^{-4}s^{-1}, & \text{x}\leq 20\;\mu \text{m}\;\text{and}\;\text{x}>=30\;\mu m\\ \gamma_{on}^{\prime }=\;5.16\times 10^{-3}s^{-1}, & x\in [20,30]\mu \text{m} \end{array}$$ (7)

The rate parameter about phosphorylation of NFs of γ_de_, *γ*_*ph*_ along the axon is shown in Fig 9. At the node of Ranvier (20μm < x < 30μm), the rate of phosphorylation (γ_ph_ = 0.1 *s*^−1^) is much lower than the rate of dephosphorylation (γ_de_ = 0.8 *s*^−1^), indicating that NFs are less phosphorylated; while in the internode section (x ≤ 20 μm and x > = 30 μm), the phosphorylation rate (*γ*_*ph*_ = 0.8 *s*^−1^) is much higher than the rate of dephosphorylation (γ_de_ = 0.1 *s*^−1^), indicating that NFs are more phosphorylated. In the constructed configuration, the model can demonstrate the effect of phosphorylation on the transport of NFs.

### 4.2 The newly developed ‘8-state’ model

In this section, we build a new model- “8-state” model by considering two extra states of phosphorylated and dephosphorylated in the “6- state” model, and derive a mathematical expression for the average velocity of NFs.

#### 4.2.1 Model description of ‘8-state’ model

Based on the experimental observations of axonal morphology and the NFs population difference between the internode and node of Ranvier [19], we assume that NFs transport decrease in the internode because of myelination and phosphorylation, while NFs in the node of Ranvier accelerate their transport, and our model increases the on-track rate from the less phosphorylated state to on-track pausing state ($\gamma_{on}^{2}$) based on the continuity equation. Our model can successfully reproduce higher NFs content in the internode and lower distribution in the node of Ranvier by 7.6 folds. In addition, by the Monte Carlo simulation and PDE solution it is true that the continuity equation holds true for the ‘8-state’ model.

Considering that NFs have transition kinetics of phosphorylation and dephosphorylation, we modify the ‘6-state’ model by subdividing two off-track pausing states at both anterograde and retrograde directions, resulting in an ‘ 8-state’ model.

**Fig 10:**
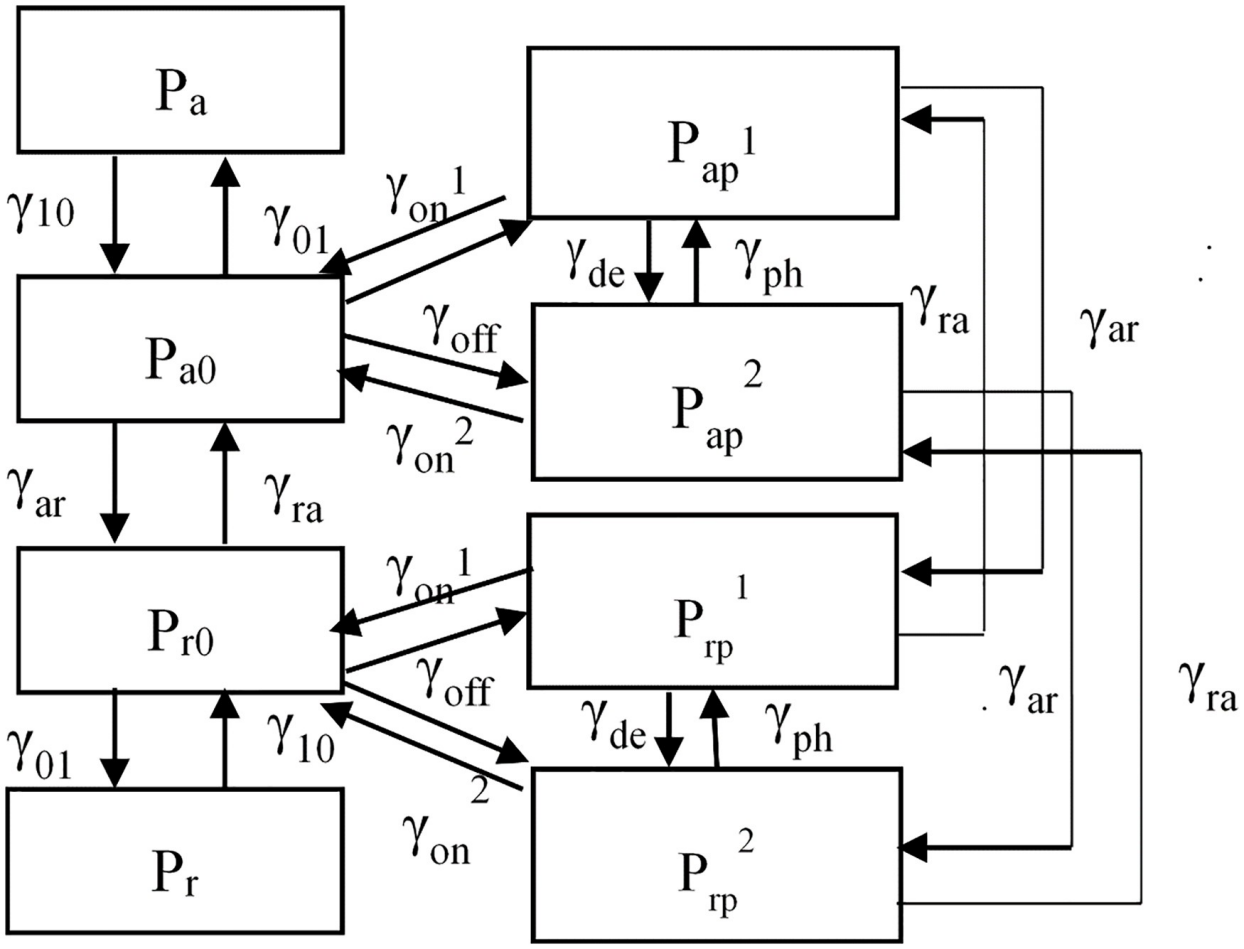
Schematic diagram of the ‘8-state’ model.

The basic assumptions of our model is that we assume only NFs at off-track states have to be divided into two sub-states, (which is different from above, where the NFs at each of the six states have both phosphorylated and dephosphorylated states): one is NFs of phosphorylation with *$P_{ap}^{1}$* and $P_{rp}^{1}$; the other is NFs of de phosphorylated with $P_{ap}^{2}$ and $P_{rp}^{2}$; and their on-track rate will be different due to the phosphorylated status, with lower value of on-track rate ($\gamma_{on}^{1}$) in phosphorylated sub-states and higher on-track rate in dephosphorylated state ($\gamma_{on}^{2}$). The schematic diagram of the transformation between the eight states is in Fig 10.

Correspondingly, the kinetic equations for NFs transport can be written as follows:

$$\begin{array}{ll} \frac{\partial {\text{P}}_{\text{a}}}{\partial t} & =-\frac{v_{a}\partial P_{a}}{\partial x}-\gamma_{10}P_{a}+\gamma_{01}P_{a0}\\ \frac{\partial {\text{P}}_{r}}{\partial t} & =-\frac{v_{r}\partial P_{r}}{\partial x}-\gamma_{10}P_{r}+\gamma_{01}P_{r0}\\ \frac{\partial {\text{P}}_{\text{a}0}}{\partial t} & =-(\gamma_{01}+\gamma_{ar}+2\gamma_{off})P_{a0}+\gamma_{10}P_{a}+\gamma_{ra}P_{r0}+\gamma_{on}^{1}{\text{P}}_{\text{ap}}^{1}+\gamma_{\text{on}}^{2}{\text{P}}_{\text{ap}}^{2}\\ \frac{\partial {\text{P}}_{\text{r}0}}{\partial t} & =-(\gamma_{01}+\gamma_{ra}+2\gamma_{off})P_{r0}+\gamma_{10}P_{r}+\gamma_{ar}P_{a0}+\gamma_{on}^{1}P_{rp}^{1}+\gamma_{on}^{2}P_{ap}^{2}\\ \frac{\partial {\text{P}}_{\text{ap}}^{1}}{\partial t} & =\gamma_{off}P_{a0}-(\gamma_{on}^{1}+\gamma_{ar}+\gamma_{de})P_{ap}^{1}+\gamma_{ra}P_{rp}^{1}+\gamma_{ph}P_{ap}^{2}\\ \frac{\partial {\text{P}}_{\text{ap}}^{2}}{\partial t} & =\gamma_{off}P_{a0}-(\gamma_{on}^{2}+\gamma_{ar}+\gamma_{ph})P_{ap}^{2}+\gamma_{ra}P_{rp}^{2}+\gamma_{de}P_{ap}^{1}\\ \frac{\partial {\text{P}}_{\text{rp}}^{1}}{\partial t} & =\gamma_{off}P_{r0}-(\gamma_{on}^{1}+\gamma_{ra}+\gamma_{de})P_{rp}^{1}+\gamma_{ar}P_{ap}^{1}+\gamma_{ph}P_{rp}^{2}\\ \frac{\partial {\text{P}}_{\text{rp}}^{2}}{\partial t} & =\gamma_{off}P_{r0}-(\gamma_{on}^{2}+\gamma_{ra}+\gamma_{ph})P_{rp}^{2}+\gamma_{ar}P_{ap}^{2}+\gamma_{de}P_{rp}^{1} \end{array}$$ (8)

The spatial distribution of NF population is the summation of all the motion states of individual NF, which can be written as

$$P(x,t)=P_{a}(x,t)+P_{r}(x,t)+P_{a0}(x,t)+P_{r0}(x,t)+P_{ap}^{1}(x,t)+P_{ap}^{2}(x,t)+P_{rp}^{1}(x,t)+P_{rp}^{2}(x,t)$$

If NFs enter the axon at the proximal end and exit the axon at a constant rate, the equilibrium distribution of NFs can be reached, and can be obtained readily by solving the above Eq (8). The Eq (8) approximates Gaussian distribution. After derivation, we can obtain the similar formula for the average velocity and steady state distributions as in Eq (2), but with a different q_2_:

$$\bar{v}=\frac{1}{\left(1+{\text{q}}_{1}(1+{\text{q}}_{2}))(1+{\text{q}}_{3}\right)}(v_{a}+q_{3}v_{r})$$ (9)

$$q_{2}=\frac{2\gamma_{off}}{\alpha_{1}\gamma_{on}^{1}+\alpha_{2}\gamma_{on}^{2}},\;where\;\alpha_{1}=\frac{\gamma_{ph}}{\gamma_{ph}+\gamma_{de}\;},\;\alpha_{2}=\frac{\gamma_{de}}{\gamma_{ph}+\gamma_{de}\;}$$

$${\;P_{ap}=P_{ap}^{1}+P_{ap}^{2};\;where,\;P}_{ap}^{1}=\alpha_{1}P_{ap},\;{\;P}_{ap}^{2}=\alpha_{2}P_{ap\;}$$

$${\;P_{rp}=P_{rp}^{1}+P_{rp}^{2};\;where,\;P}_{rp}^{1}=\alpha_{1}P_{rp},\;{\;P}_{rp}^{2}=\alpha_{2}P_{rp}$$

#### 4.2.2 Velocity reconstruction for the node and internode in ‘8-state’ model

The reproduction of NFs distribution in node and internode can readily adopt the strategy of Continuity Equation [14], since we have already derived the analytical expression of average velocity $\overset{-}{v}$ in Eq (9). It can be exemplified by reconstruction of the on-track rate in node and internode respectively, and then numerically solving the partial differential Eq (8) to reproduce the distribution of NFs along axon for both node and internode.

The on-track rate $\gamma_{on}^{2}$ at node and internode are reconstructed by the average velocity Eq (9), by keeping the on track rate $\gamma_{on}^{1}$ unchanged $\gamma_{on}^{1}=2.75\times 10^{-4}s^{-1}$, then we have:

$$\gamma_{on}^{2}=\frac{\frac{2\gamma_{off}}{q_{2}}-\alpha_{1}\gamma_{on}^{1}}{\alpha_{2}}\;;\;where\;{\text{q}}_{2}=\frac{\left[(v_{a}+q_{3}v_{r})/((1+q_{3})\bar{v})-1\right]}{q_{1}}-1\;.$$ (10)

For the internode, the average velocity is assumed to be ${\overset{-}{v}}_{internode}=1.01\;mm/day$, therefore, in the internode the rate parameters are: $\gamma_{on}^{2}=5.16\times 10^{-3}s^{-1}$; the phosphorylation rates are set as before *γ*_*ph*_ = 0.8 *s*^−1^, *γ*_*de*_ = 0.1 *s*^−1^.

**Fig 11:**
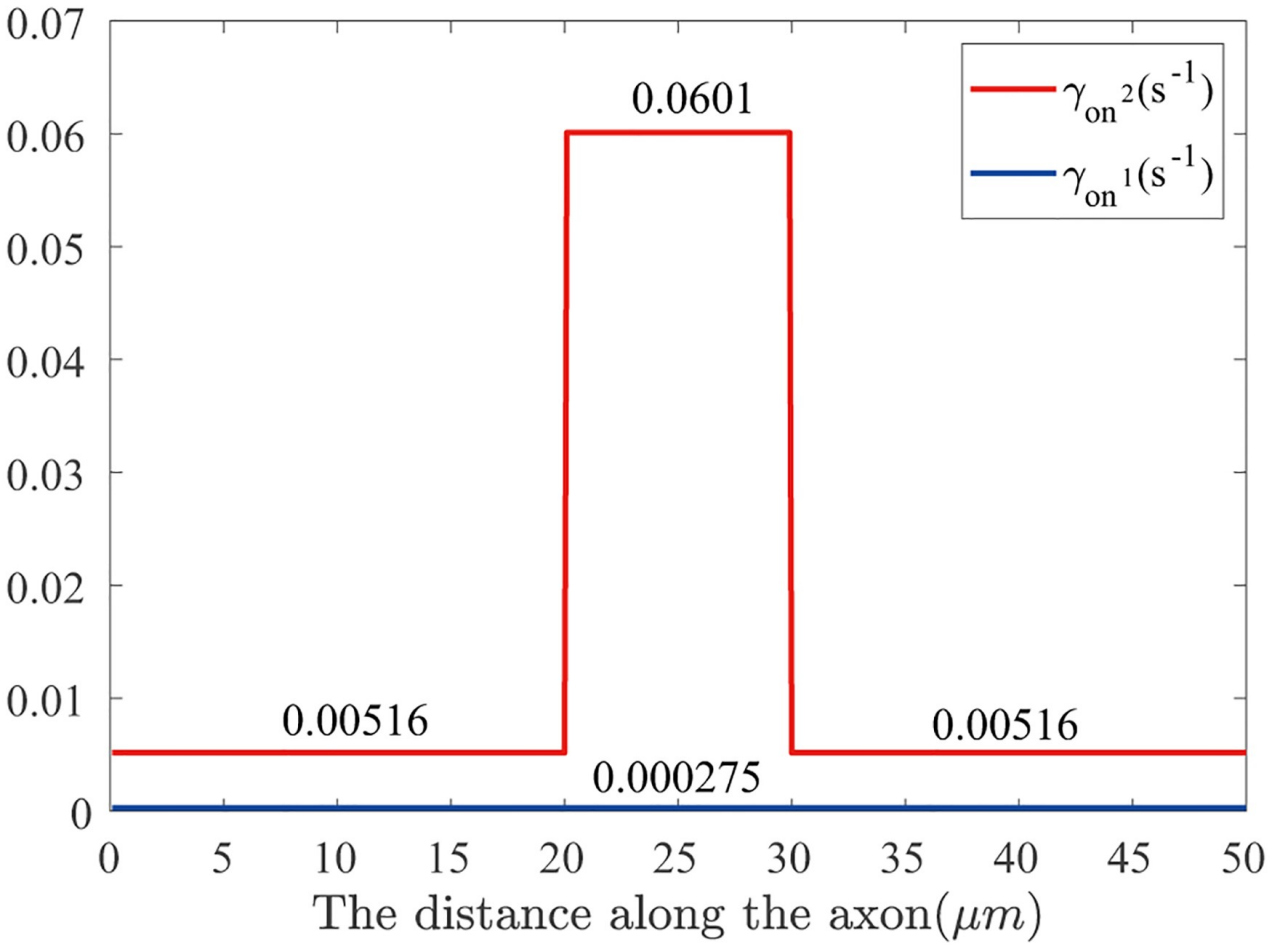
Distribution of on-track rates $\gamma_{on}^{1},\;\gamma_{on}^{2}$ along node and internode for ‘8-state’ model.

While at node of Ranvier, the average velocity is ${\overset{-}{v}}_{node}=7.6\times {\overset{-}{v}}_{internode}=7.69\;mm/day$, therefore in the node of Ranvier, the on-track rate of $\gamma_{on}^{2}=6.01\times 10^{-2}s^{-1}$. And the phosphorylation rates are set as before *γ*_*ph*_ = 0.1*s*^−1^, *γ*_*de*_ = 0.8 *s*^−1^. The distribution of on-track rates along axon can be seen in Fig 11.

By the analytical calculation in Eq (9), NFs population distribution at each state of equilibrium in the internode can be found:

$$p_{a}=2.85\times 10^{-2},p_{r}=8.5\times 10^{-3},\;p_{a0}=6.27\times 10^{-2},\;p_{r0}=1.87\times 10^{-2},p_{ap}^{1}=0.603,p_{ap}^{2}=0.0754,\;p_{rp}^{1}=0.1809,\;p_{rp}^{2}=0.0226.$$

And we can calculate the average velocity ${\overset{-}{v}}_{internode}=0.0117\mu m/s;$

For NFs at node of Ranvier, the distribution of NFs at equilibrium in each state is calculated, and the result is: *p*_*a*_ = 2.17 × 10^−1^, *p*_*r*_ = 6.05 × 10^−2^, *p*_*a*0_ = 4.74 × 10^−1^, *p*_*r*0_ = 1.42 × 10^−1^, $p_{ap}^{1}=8.8\times 10^{-3},\;p_{ap}^{2}=0.0701,\;p_{rp}^{1}=0.0026,\;p_{rp}^{2}=0.0210.$ And the average velocity is ${\overset{-}{v}}_{node}=0.0890\mu m/s$.

Therefore, the average velocity of NFs at node and internode is the same as we constructed in the beginning: $\frac{{\overset{-}{v}}_{node}}{{\overset{-}{v}}_{internode}}=\frac{0.0890\mu m/s}{0.0117\mu m/s}=7.6$. The PDE simulation result is the same as the analytical calculations, as can be seen in the Fig 3.

## Supporting information

S1 Data (RAR)
